# Multispectral Light Detection and Ranging Technology and Applications: A Review

**DOI:** 10.3390/s24051669

**Published:** 2024-03-04

**Authors:** Narges Takhtkeshha, Gottfried Mandlburger, Fabio Remondino, Juha Hyyppä

**Affiliations:** 1 3D Optical Metrology (3DOM) Unit, Bruno Kessler Foundation (FBK), 38123 Trento, Italy; ntakhtkeshha@fbk.eu; 2 Department of Geodesy and Geoinformation, Vienna University of Technology, 1040 Vienna, Austria; gottfried.mandlburger@geo.tuwien.ac.at; 3 Department of Photogrammetry and Remote Sensing, Finnish Geospatial Research Institute, National Land Survey of Finland, FI-02150 Espoo, Finland; juha.hyyppa@nls.fi

**Keywords:** multispectral laser scanning, point clouds, intensity, hyperspectral LiDAR, sensors

## Abstract

Light Detection and Ranging (LiDAR) is a well-established active technology for the direct acquisition of 3D data. In recent years, the geometric information collected by LiDAR sensors has been widely combined with optical images to provide supplementary spectral information to achieve more precise results in diverse remote sensing applications. The emergence of active Multispectral LiDAR (MSL) systems, which operate on different wavelengths, has recently been revolutionizing the simultaneous acquisition of height and intensity information. So far, MSL technology has been successfully applied for fine-scale mapping in various domains. However, a comprehensive review of this modern technology is currently lacking. Hence, this study presents an exhaustive overview of the current state-of-the-art in MSL systems by reviewing the latest technologies for MSL data acquisition. Moreover, the paper reports an in-depth analysis of the diverse applications of MSL, spanning across fields of “ecology and forestry”, “objects and Land Use Land Cover (LULC) classification”, “change detection”, “bathymetry”, “topographic mapping”, “archaeology and geology”, and “navigation”. Our systematic review uncovers the potentials, opportunities, and challenges of the recently emerged MSL systems, which integrate spatial–spectral data and unlock the capability for precise multi-dimensional (nD) mapping using only a single-data source.

## 1. Introduction

LiDAR is a renowned and widely used technology [1]. Fast and accurate acquisition of 3D information is the primary advantage of this 3D surveying technology. Laser sensors can be mounted on or carried by several platforms: crewed and uncrewed airborne, satellite, terrestrial, and mobile (including hand-held, backpack, and vehicle-based LiDAR). Additionally, LiDAR data play an important role in the generation of 3D models ranging from cities and other sites, Digital Surface Models (DSMs), and Digital Terrain Models (DTMs). LiDAR technology has evolved dramatically since its appearance in the late 90s. One of the latest and rapidly developing achievements in laser scanning technology is multispectral LiDAR and hyperspectral LiDAR (HSL) systems, having the ability to concomitantly obtain both geometrical and spectral information of the surveyed scene [2]. The utilization of intensity information, in conjunction with LiDAR’s geometric data, has enabled the extraction of additional features that could serve various purposes in remote sensing and photogrammetry. While passive multi/hyperspectral images have actively shown satisfactory results for land use and land cover classification as well as target detection, their lack of 3D information limits their capabilities, especially for interpreting complex scenes. Even though LiDAR data can be combined with multi/hyperspectral passive images to improve scene characterization [3,4,5], registration problems in space (i.e., alignment and resolution) as well as time (i.e., changing observation conditions and dynamic scene), make this fusion challenging [6]. This drawback has prompted the development of MSL and HSL scanners as single-data source solutions to simultaneously acquire 3D geometric and radiometric information and alleviate the mentioned problems. For these reasons, MSL systems are currently gaining interest. Compared to conventional monochromatic (single-wavelength) LiDAR data, MSL data ensure a higher level of reliability and accuracy in object detection and scene classification [7,8,9,10,11,12,13,14,15]. Furthermore, MSL technology is able to acquire numerous textures of targets [16]. Additionally, delineation of individual trees is quite often difficult when only geometric spatial information of LiDAR data is taken into account [17]. Notably, contrary to optical imagery sensors, LiDAR is an active remote sensing sensor that has independent data acquisition of external illumination conditions, perfectly addressing the common shadow issue in the processing of optical images. Moreover, as reported by the results in [18,19], MSL data can dramatically enhance object detection in comparison to multispectral images. That is why MSL is becoming a popular source of research data for nD mapping, considering the intensities information as further dimensions.

### 1.1. Paper’s Contribution

A MSL technology offers numerous opportunities for remote sensing and photogrammetric applications. However, a comprehensive literature review on this emerging technology is currently lacking. Therefore, this study aims to fill this gap by providing a detailed overview on the current state-of-the-art of MSL technology and its applications. To propose a comprehensive roadmap for the comprehension and exploitation of MSL systems, this review paper focuses on the latest advancements in multispectral sensor technology, with a special highlight on the recent technology and applications in the domain of MSL. Essentially, this study addresses the following research inquiries:

- What is the historical evolution, current status, and prospective future of MSL technology?
- What advantages do MSL data offer in comparison to multispectral images as well as monochromatic LiDAR data?
- What categorizations exist for MSL data, and what are the inherent potentials and challenges associated with each?
- Are there established benchmark datasets available for MSL?
- What is the scope of the application of MSL within the fields of remote sensing and photogrammetry?
- What are the prospective benefits, opportunities, and challenges linked with MSL technology?

**Figure 1:**
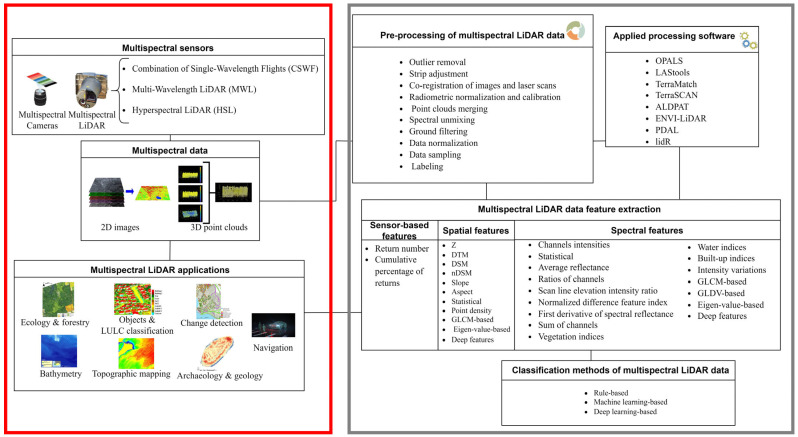
Summary of the current state-of-the-art in MSL technology. This paper focuses on the left side of the figure, highlighted in red.

The historical development and recent technological advances for the acquisition of multispectral (MS) data are outlined in Section 2. Section 3 reviews the existing literature on MSL data types and benchmarks. Several applications, organized per research domains, are reported in Section 4. Potentials, opportunities, and challenges of MSL are discussed in Section 5. Finally, Section 6 draws some conclusions and outlines new perspectives for future studies and developments. Figure 1 provides a summary of the presented state-of-the-art in MSL technologies and processing steps. It is worth noting that the processing aspects (grey part) of MSL data are not in the scope of this article and will be presented in following review papers by the same authors. A detailed summary of reviewed studies on MSL data is given in Table A1 of Appendix A.

### 1.2. Literature Search Strategy

To conduct a comprehensive literature review and present the findings, we initiated the process by formulating a search query using two widely recognized academic databases, namely Scopus [20] and Web of Science [21]. The details of this initial screening are reported in Table 1. The search query was designed as a combination of two distinct parts. The first part encompassed terms of “multispectral”, “multi-wavelength”, “bispectral’’, “hyperspectral”, and “dual-wavelength”, which denote the multispectral nature of the data, while the second parts refer to LiDAR instruments. Subsequently, we applied a set of exclusion criteria to refine the results. Articles that did not directly contribute to remote sensing and photogrammetry or failed to validate the proposed study or were older than 2005 were excluded from the review process. We considered seven applications of “ecology and forestry”, “objects and LULC classification”, “change detection”, “bathymetry”, “topographic mapping”, “archaeology and geology”, and “navigation”, as mentioned. Following a rigorous screening process, 89 high-quality papers were identified as suitable for in-depth analysis and review.

## 2. Multispectral Sensors

Multispectral sensors can be divided into two main groups: passive (optical) and active sensors. A comparison of multispectral sensors is reported in Table 2. Basically, passive sensors rely on and are affected by environmental illumination. Conversely, MSL sensors mostly have lower coverage, as they are capable of mitigating the environmental illumination influence on spectral information collection [22]. Moreover, passive sensors have a limited capacity to penetrate objects (e.g., vegetation, water surface) [23]. Even when stereo imagery is acquired, it is very difficult to determine the depth of water columns and derive nautical information, such as lake volume, channel cross-sections, and seabed properties. Furthermore, multispectral optical sensors typically produce two-dimensional images.

**Figure 2:**
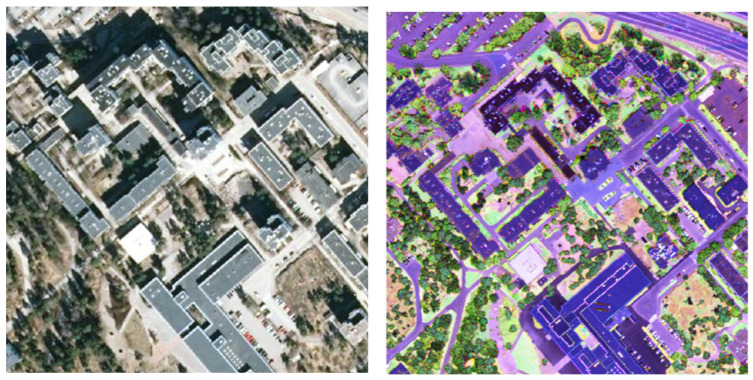
Aerial optical image (**left**) versus MSL-based false-color image (**right**), Optech Titan LiDAR [16].

In conjunction with 2D data, the shadowing effect and relief displacement are generally drawbacks of optical imagery, causing processing issues and even misinterpretations [24]. Anisotropy caused by directional reflectance combined with varying view angles presents a challenge to automatic classification, particularly when passive optical sensors are used [25]. On the other hand, MSL sensors provide information on the full 3D distribution of materials with improved penetration capacity. This is the main advantage, especially for detecting semi-transparent objects such as vegetation and fences. Laser scanning also provides clear information in shadowed areas, which is another advantage of MSL over multi/hyperspectral images (see Figure 2). This merit is particularly significant in urban areas with high buildings, which are the primary cause of shadows on aerial images. As the recorded spectral values in passive optical sensors depend on sunlight conditions, the spectral values for a specific object may not be comparable between different images, making change detection cumbersome. Understanding LiDAR intensity is discussed in [26]. Using MSL, Kukkonen et al. [12] found that it provided comparable accuracy to monochromatic LiDAR data combined with aerial imagery for predicting dominant tree species, capable of distinguishing between conifers and broadleaved trees more precisely. Also, they concluded that MSL data performed less well in predicting species-specific volume models than monochromatic or MSL data combined with aerial images. Laser scanner sensors also have the advantage of separating tree canopy and ground data, whereas passive optical sensors often offer mixed signals [27]. While we are witnessing continuous advancements coming from computer vision and robotics to support the processing of multispectral optical images, unfortunately, there is still limited understanding of MSL data. Indeed, modern processing techniques, especially deep learning (DL) architectures, developed for this type of data, are highly limited compared to conventional raster-based DL models for processing MS images.

### 2.1. Multispectral Passive Sensors

Table 3 provides a list of frequently used multispectral sensors. Basically, multispectral passive sensors provide 2D images from the scene. Furthermore, digital optical sensors and photogrammetric software enable the creation of extensive 3D point clouds through the matching of stereo aerial images automatically. It is worth noting that while photogrammetric point clouds and MSL serve as single-data source options for acquiring both geometric and radiometric information, a study conducted by Kukkonen et al. [28] showcased the notable superiority of MSL over photogrammetric point clouds in the prediction of boreal tree species’ volumes. This superiority is attributed to MSL’s ability to provide more detailed structural information compared to photogrammetric-based data [28].

### 2.2. Multispectral Laser Systems

**Figure 3:**
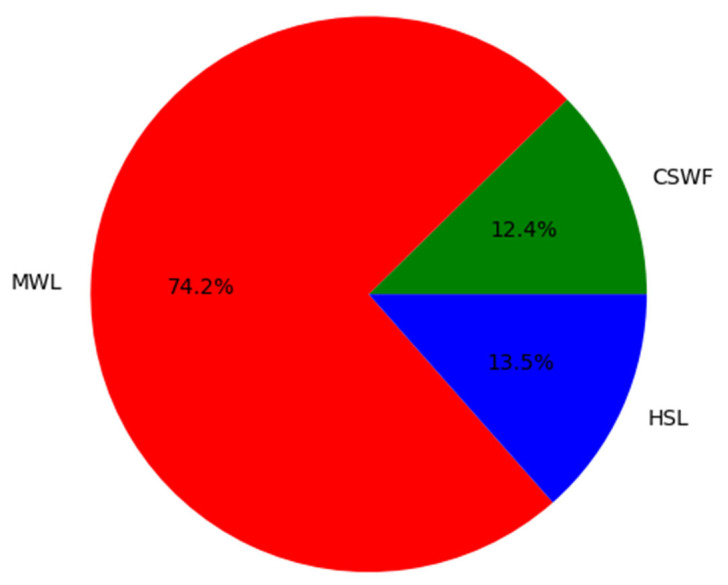
Distribution of employed MSL systems in the literature—based on Table A1.

MSL sensors with the capability of 3D data acquisition in two or more wavelength channels are among the leading advances in 3D data collection. Sensor properties (e.g., wavelength, instrument size, and measurement range) are selected with respect to the intended application, causing instruments with different wavelengths. The power of some laser wavelengths and their eye safety make it difficult to apply them to long-range airborne laser scanning (ALS) [39]. Considering human eye sensitivity to visible wavelengths, it is safer to have multiple laser scanner channels in the 1.3–1.6 micrometre range. The majority of monochromatic ALS systems operate in the near-infrared (NIR) region of the electromagnetic spectrum due to the fact that most topographic features reflect NIR in sufficient amounts required for registration on the receiver [39,40]. According to the literature review, 532 nm (green), 1064 nm (NIR), and 1550 nm (short-wavelength infrared, SWIR) are the most commonly available laser wavelengths in ALS [24]. In the case of terrestrial or UAV-based laser scanners, Velodyne and Ouster are widely used sensors, operating primarily in the wavelength range around 900 nm [41]. Available dual-wavelength laser scanners are mostly hydrographic and bathymetric systems for coastal zone and shallow water mapping, which are equipped with green and sometimes with red and infrared wavelengths in addition to the green channel. In all laser scanner systems, for eye safety reasons, the beam divergence of the green wavelength (0.7–1.0 mrad) is usually greater compared to NIR and SWIR laser scanners (approximately 0.2–0.5 mrad), leading to a larger footprint at the same distance from the sensor. However, because of the mentioned footprint sizes, information from individual wavelengths is even more complementary. Nevertheless, co-registration problems are likely to arise when combining the data positions [42]. Additionally, modern MSL systems emit different wavelengths at different angles, resulting in separate scan lines and differences in the positions of measurements. Comparing spectral profiles derived from MSL/HSL has revealed that this cutting-edge technology is capable of collecting spectral information as trustable as laboratory measurements using spectrometers [43,44]. MSL data acquisition techniques can generally be divided into three approaches: Combination of Single-Wavelength Flights (CSWF), Multi-Wavelength LiDAR (MWL), and HSL systems, operating at different wavelengths. In the following subsections, the mentioned techniques are discussed in detail. Figure 3 illustrates the distribution of employed MSL systems in the literature, categorized according to their types. This analysis is based on a comprehensive review of 89 technical papers (see Table A1). As shown, MWL emerges as the predominant category (74.2%) within MSL systems, while HSL and CSWF respectively exhibit comparatively lower utilization rates.

#### 2.2.1. Combination of Single-Wavelength Flights (CSWF)

Combining several independent single-wavelength flight missions is one of the common approaches of MSL data acquisition, undertaken in attempts to overcome pre-existing technical and commercial constraints. In order to acquire MSL data, Briese et al. [45] exploited three monochromatic airborne laser scanners, namely RIEGL VQ-820-G [46], RIEGL VQ-580 [47], and RIEGL LMS-Q680i [48], operating at the respective laser wavelengths of 532 nm, 1064 nm, and 1550 nm. In another study, Junttila [49] utilized three terrestrial laser scanners: Leica HDS6100 [50], FARO S120 [51], and FARO X330 [52], each operating at distinct wavelengths of 690 nm, 905 nm, and 1550 nm, respectively. In addition to MSL, thanks to the ongoing development of laser scanning techniques, various types of LiDAR sensors have become available during recent years, entailing single-wavelength Linear Mode LiDAR (LML), Single Photon LiDAR (SPL), Geiger Mode LiDAR (GML), Full Waveform Digitization (FWD) LiDAR, and Multi-Pulse in Air (MpiA) LiDAR [24]. The sensitivities of SPL and GML to single photons distinguish them from conventional LML. Moreover, sensitive SPL systems are capable of being operated at higher flying altitudes, making them efficient for data acquisition over large areas and country-wide mapping [53]. Despite the high sensitivity and wide coverage of monochromatic LiDAR sensors, some researchers have discovered significant confusion between ground-level objects (e.g., low vegetation, asphalt, gravel, and rock areas) when they investigated their potential for land cover classification [54,55]. Motivated by this issue, Matikainen et al. [55] explored the potential of combining the information from the first and second channels of the multispectral Titan laser scanner with SPL and obtained promising results. Therefore, their work can be considered the next step in this type of MSL data capturing. Table 4 shows some monochromatic LiDAR sensors that would be useful for generating MSL datasets.

#### 2.2.2. Multi-Wavelength LiDAR (MWL)

**Figure 4:**
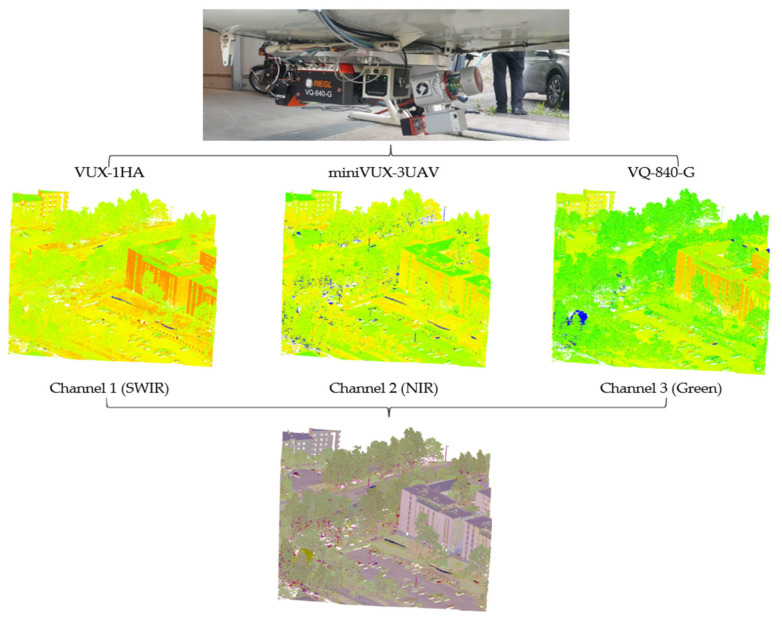
Operational mechanism of multispectral LiDAR data acquisition by the “HeliALS-TW” MSL system.

Further developments in the mounting capabilities of aircrafts and other platforms have made the acquisition of MSL data possible by integrating distinct monochromatic LiDAR sensors simultaneously mounted on the same platform. Figure 4 depicts the operational mechanism of multispectral LiDAR data acquisition by MSL systems. In effect, this figure shows a multispectral point cloud gathered by the “HeliALS-TW” MSL (developed by the Finnish Geospatial Research Institute (FGI)), which is a composite of three monochromatic RIEGL laser scanners bolted together, namely, the VUX-1HA, miniVUX-3UAV, and VQ-840-G, acting at wavelengths of 1550 nm, 905 nm, and 532 nm, respectively [68]. Merging the point clouds of these three mentioned channels results in multispectral point cloud data.

In 2006, the federal institute for materials research and testing (BAM) developed the first four-wavelength airborne MSL to take advantage of such systems for the inspection of building surfaces [69]. The Salford Advanced Laser Canopy Analyser (SALCA) is the first multispectral full waveform terrestrial LiDAR, designed in 2010 for characterizing forest canopies [70]. This experimental MSL performs in two wavelengths in the near and middle-infrared (1040 and 1550 nm). In 2012, Boston University developed a full-waveform terrestrial dual-wavelength MSL named DWEL (Dual-Wavelength Echidna LiDAR) for the automated retrieval of forest structure [71]. This dual-wavelength MSL functions at 1064 nm and 1548 nm. In the same year, Wei et al. [72] designed an MSL system for vegetation applications, called Multi-Wavelength Canopy LiDAR (MWCL). This terrestrial MSL operates with four lasers of different wavelengths chosen according to nitrogen stresses that make changes in the spectral reflectance of rice leaves. Their experimental results demonstrate the high capability of recording the physiology of the canopy, which is not possible when solely employing traditional monochromatic LiDAR. Moreover, to obtain three-wavelength LiDAR data, Briese et al. [73] conducted two flight missions with the same flight plans within four days in 2013. In the first mission, a RIEGL VQ-820-G and RIEGLVQ-580 were combined, whereas a RIEGL VQ-820-G and RIEGL VQ-480i were employed in the second mission.

Lindberg et al. [74] utilized a dataset collected similarly to [73]. According to their report, while the proportion of returns at different heights above the ground and the level of detail almost resembled, the point density of RIEGL VQ-820-G data was slightly higher than the other two wavelengths. Furthermore, as the RIEGL VQ-820-G scanner is principally designed for bathymetric mapping, it has a higher scanning sensitivity than the two other scanners. Moreover, the green wavelength of the RIEGL VQ-820-G sensor has twice as much angular divergence, which leads to a larger footprint, density, and noise. Optech Titan is the first commercial MSL that was launched in 2014 by Teledyne Optech (Vaughan, ON, Canada). A Titan LiDAR system is able to simultaneously capture spectral information at three channels with wavelengths of 1550 nm (C1 = SWIR), 1064 nm (C2 = near infrared, NIR), and 532 nm (C3 = green), at different looking angles of 3.5° forward, 0° nadir, and 7° forward, respectively. This sensor acquires three separate point clouds. Detailed specifications of Optech Titan MSL can be found in [75] and Table 5. Regarding the reflectance behavior of different wavelengths while interacting various ground objects, the green channel of the Titan sensor allows for shallow water mapping, while the second channel (NIR) is beneficial for detecting vegetation. Furthermore, soil identification can be facilitated by utilizing SWIR and green channels. During recent years, the performance of Optech Titan multispectral data has been extensively explored in varied applications such as land cover classification, forest mapping, water depth measurement, etc. (see Table A1). Recently, the RIEGL company has launched a dual-wavelength system, namely RIEGL VQ-1560i-DW [76] on the market, involving two wavelengths of 532 nm and 1064 nm. This laser scanner has been successful in mapping vegetation and agriculture by providing the capability of calculating the Green Normalized Difference Vegetation Index (GNDVI).

The combination of the previously mentioned MSL data acquisition approaches was employed and compared by Hopkinson et al. [77] with three ALS flight missions over two years to characterize and classify a forest environment. The first one was conducted by Aquarius (532 nm) and Orion (1550 nm) sensors co-mounted in a Piper Chieftan survey aircraft. The Gemini (1064 nm) sensor was deployed for the second flight, and the Titan sensor was applied in the last mission. As reported by their results, the multispectral Titan sensor dramatically surpassed MSL data capturing through the CSWF approach. Gong et al. [78] successfully developed a four-wavelength (556 nm, 670 nm, 700 nm, and 780 nm) ground observation MSL system for remote sensing classification and monitoring of vegetation. Similarly, Woodhouse et al. [79] and Wallace et al. [80] developed a set of four-wavelength MSL systems for vegetation information extraction. In 2021, Teledyne Optech unveiled its latest MSL, named Coastal Zone Mapping Imaging LiDAR (CZMIL) Supernova, representing a dual-wavelength airborne MSL, specifically designed for topo-bathymetry scanning applications. A summary of some practical and experimental MSL systems is presented in Table 5. It is noteworthy that unlike the airborne MSL, all terrestrial MSL scanners are still experimental, and no commercial instrumentation is yet available. A comprehensive review of terrestrial MSL laser scanners can be found in [81].

**Table 5:** List of some developed MSL sensors. M = mobile; C = channel; RR = range resolution; PD = point density; NA= not available.

LiDAR Sensor	Producer	Wavelength [nm]	Main Application	Beam Divergence [mrad]	Looking Angle [°]	PRF [kHz]	PD [points/m^2^]
Optech Titan, A	Teledyne Optech	C1: 1550 C2: 1064 C3: 532	Multi-purpose	C1: 0.35 C2: 0.35 C3: 0.7	C1: 3.5 C2: 0 C3: 7	900	Bathymetry: >5 pts/m^2^ Topography: >45 pts/m^2^
HeliALS-TW, A	FGI	C1: 1550 C2: 905 C3: 532	Forest inventory	C1: 0.5 C2: 0.5 × 1.6 C3: 1	C1: 360 C2: 120 C3: 28 × 40	C1: 1017 C2: 300 C3: 200	C1: 1400 C2: 500 C3: 1600
HawkEye-5, A [82]	Leica	C1: 515 C2: 515 C3: 1064	Deep and shallow bathymetry & topography	C1: 7.5 C2: 4.75 C3: 0.5	±14 front/back ±20 left/right	C1:40 C2: 200 C3: 500,000	C1: 1 C2: 5 C3: 12
VQ-880-GH, A [83]	RIEGL	C1: 532 C2: 1064	Deep and shallow bathymetry & topography	0.7–2	40	C1: 700 C2: 279	NA
CZMIL Supernova, A [84]	Optech	C1: 532 C2: 532 C3: 1064	Deep and shallow bathymetry & topography	7	40	C1:30 C2: 210 C3: 240	Shallow water ≤ 8 Deep water ≥ 1
VQ-1560i-DW, A	RIEGL	C1: 532 C2: 1064	Agriculture & forestry, bathymetry	C1: 0.7–2 C2: 0.18–0.25	14	1000	2–60
Chiroptera4X, A [85]	Leica	C1: 532 C2: 1064	Bathymetry & topography	~3	±14 front/back, ±20 left/right	140 500	Bathymetry: >5 Topography: >10
DWEL, T	Boston University	C1: 1064 C2: 1548	Forest inventory	1.25, 2.5, or 5	±119 front/back ±119 left/right	20	NA
BAM, T	BAM	C1: 670 C2: 810 C3: 980 C4: 1930	Inspection of building surfaces	NA	30	10,000	NA
MWCL, T	Wuhan University	C1:555 C2: 670 C3: 700 C4: 780	Vegetation mapping	C1: 0.3 × 0.6 C2: 0.3 × 0.6 C3: 0.2 × 0.6 C4: 0.2 × 0.6	25	0.8	NA

#### 2.2.3. Hyperspectral LiDAR (HSL)

In contrast to passive sensing, which distinguishes between multispectral and hyperspectral just based on the number of channels used, LiDAR sensing relies solely on the light source as the differentiating factor. In fact, most of LiDAR sensors utilize a supercontinuum (SC) light source and send nanosecond pulses of directional broadband light using cascaded nonlinear optical interactions in an optical fiber, referred to as HSL [42,86]. This is due to the fact that when supercontinuum light source is used, the number of received bands can be decided. On the contrary, laser scanners that use a traditional LiDAR sensor but operate at different wavelengths are known as MSL. Essentially, supercontinuum lasers are the only way to increase the number of channels and enable a hyperspectral implementation for laser scanners [87]. The effect of a supercontinuum light source in creating HSL systems is discussed in detail in a review study by Li et al. [88].

The first experimental HSL system was presented in 2007 by FGI, with six wavelengths ranging from 600 to about 2000 nm [42]. The University of Maryland developed spectral LAser Detection And Ranging (LADAR) in 2011, operating across 25 spectral channels (1080–1620 nm) [89]. In 2012, FGI designed a full waveform HSL with eight spectral channels for terrestrial laser scanning [86]. This laser scanner performs at the spectral range of 480–2200 nm and produces 1 ns pulse at a repetition rate of 24 kHz. Wallace et al. [43] proposed a prototype HSL system, leveraging a super-continuum laser source to have four laser wavelengths in conjunction with Time-Correlated Single Photon Counting (TCSPC) receiver technology, which harnesses the advantages of improved depth resolution and sensitivity of the TCSPC technique. In addition, to enhance the spectral resolution of HSL scanners, innovative Acoustic–Optical Tuneable Filter-based terrestrial HSL systems (AOTF-HSL) have recently been proposed [22,90,91,92,93,94]. In such laser scanners, AOTF acts as a spectral bandpass filter on the outgoing laser from the super-continuous laser in the emission unit. The developed AOTF-HSL systems have different numbers of wavelengths, from eight [95] to 91 channels [87]. So far, prototype AOTF-HSL systems have been designed for a variety of applications, including aiding in point cloud matching in SLAM [93], vegetation red edge parameter extraction [22], coal/rock classification [87], wood–leaf separation [91], and point cloud classification [92]. Most developed HSLs obtain spectral information in the visible and near-infrared ranges (400–1000 nm) [44]. To take advantage of the longer wavelength range, which has shown more promising results for classification and detection (e.g., vegetation water content), Sun et al. [44] proposed an eight-channel HSL covering visible, NIR, and even SWIR (450–1460 nm). For further information on HSL technology, readers are referred to [96].

#### 2.2.4. Historical Development of Multispectral LiDAR

The historical evolution of MSL merits examination from two perspectives: (i) experimental vs. industrial MSL systems and (ii) development of MSL systems with more than two wavelengths. Based on our review, BAM [69] emerges as the pioneering MSL, conceptualized in 2006. This experimental terrestrial MSL is specifically crafted for the inspection of building surfaces, performing with four wavelengths (670–1930). With the introduction of the SC light source, the first experimental HSL was developed in 2007 by FGI as the next generation of MSL. In 2010, the first full waveform MSL, named SALCA [70], was experimentally devised for the purpose of forest mapping. This MSL is dual-wavelength and terrestrial. Since 2010, substantial and ongoing efforts have been dedicated to the introduction of new MSL and HSL systems such as MWCL and HeliALS-TW, with the focus primarily on increasing spectral resolution by incorporating TCSPC receiver technology and developing AOTF-based MSL/HSL (refer to Section 2.2.2 and Section 2.2.3).

Concerning commercial MSL systems, Optech Titan was the first manufactured system in 2014. Operating across three wavelengths, this airborne MSL has received substantial attention for its efficacy in diverse applications (see Table A1). After the Optech Titan, RIEGL VQ-1560i-DW, RIEGL VQ-880-GH, Leica Chiroptera4X, CZMIL Supernova (Teledyne Optech), and HawkEye-5 are dual-wavelength industrial MSL sensors introduced in 2017, 2018, 2021, and 2023, respectively. Since they act in the green and NIR spectrum, they are predominantly considered as bathymetric multispectral scanners.

## 3. Multispectral LiDAR Data

**Figure 5:**
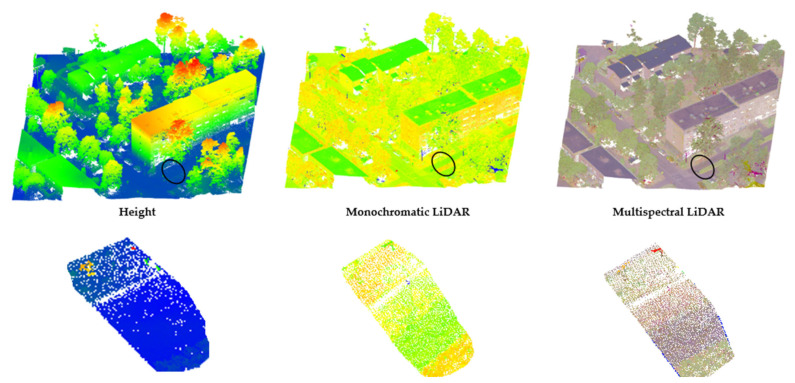
Multispectral versus monochromatic LiDAR data.

In general, compared to conventional monochromatic LiDAR, MSL is more profitable in classifying ground-level classes such as asphalt and low vegetation. The reason is that identifying elevated objects, such as buildings, trees, and powerlines, is more geometrically based, whereas ground-level objects all have a similar geometric structure, and detection must primarily rely on spectral information [16]. Figure 5 illustrates the superiority of MSL data over monochromatic LiDAR. In the zoomed area, three ground-level objects—road, soil, and grass—are entirely indistinguishable based on height values alone, as they fall within the same height range. Moreover, even though a single intensity channel may aid in identifying these classes, it still remains challenging, particularly in distinguishing soil and grass due to the lack of significant contrast between their values. In contrast, multispectral LiDAR data distinctly reveals these objects, addressing the limitations encountered with monochromatic LiDAR. The high potential of MSL systems in discriminating between various types of unique land covers, including three types of asphalt, two types of roof materials, and two types of soil, is proven by Ekhtari et al. [15] and Matikainen et al. [16].

**Figure 6:**
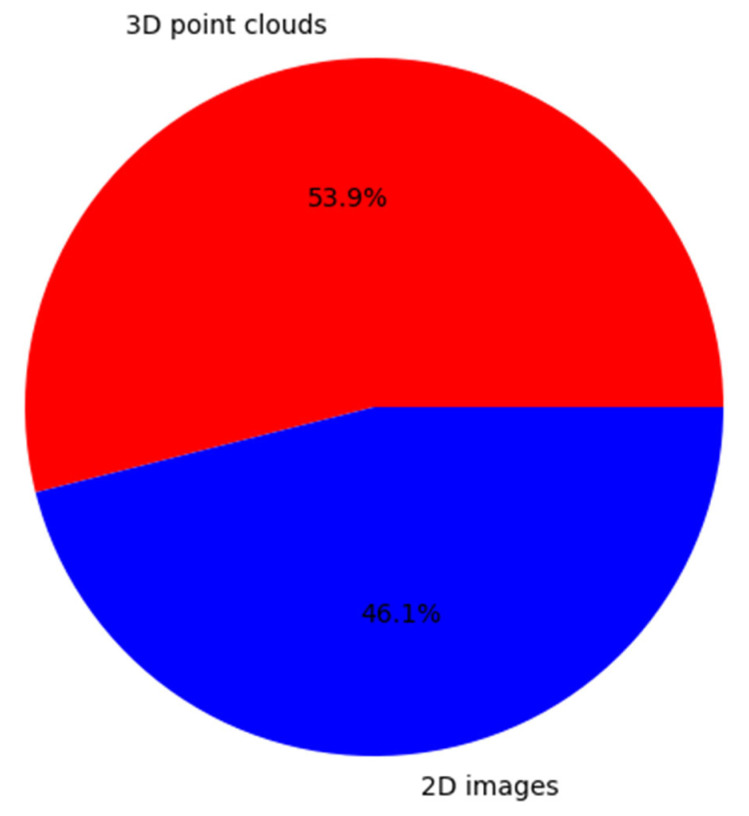
Percentage of the used MSL data type among the reviewed studies presented in Table A1.

In terms of data format, MS data can be grouped into rasterized 2D images and 3D point clouds. The development of LiDAR technologies has led to higher point density. More importantly, since MSL provides individual point clouds for each spectral channel, the data volume is substantial. While point clouds have richer 3D spatial information than images and thus describe the features of objects in a manner closer to reality, they are unstructured and irregularly distributed. That is why unstructured point cloud data have been transformed into structured data by voxelization or projection in a noticeable number of studies. As well as being time-efficient when processing large-scale MSL data, two-dimensional interpolated MSL data have the benefit of employing established image processing techniques. Even so, data conversion by interpolation of 3D point clouds brings quantization errors and spatial information loss. Consequently, directly processing 3D multispectral point clouds has become much more attractive and has made significant progress. A number of studies have demonstrated that the direct point-wise classification of multispectral data outperforms the common approach of rasterizing the point cloud prior to processing [3,93,97]. For instance, 2D surface models generally provide favorable results for detecting a large fraction of the tallest dominant trees, although methods utilizing the whole 3D point cloud data are needed for detecting suppressed smaller trees [68,98]. With the aim of reducing mixed species classes, Lindberg et al. [99] proposed a method in which raster cells are smaller (0.5 m) than what has previously been used (i.e., a typical size of 15 m × 15 m) and extracted the intensity-based features inside small raster cells using a moving window average approach. In this approach, to ensure that information from every channel is always present in every raster cell, the size of each raster cell was chosen to be large enough. As summarized in Figure 6, the majority of conducted research on MSL technology (53.9%) is based on 3D point clouds.

### MSL Benchmark Datasets

**Figure 7:**
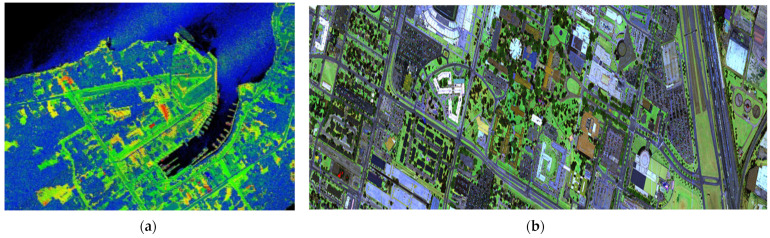
MSL benchmark datasets: (**a**) ISPRS WG III/5 dataset [101]; (**b**) IEEE GRSS MSL dataset [102].

So far, two public multi/hyperspectral datasets have been released within the geomatics community, facilitating the development and comparison of new algorithms for data processing. The first dataset was released in 2017 by ISPRS WG III/5 in collaboration with Teledyne Optech [100,101]. The data were collected using the Optech Titan MSL over a natural coastal area. In 2018, IEEE GRSS organized a contest on the fusion of MSL and hyperspectral images using a dataset acquired by the National Center for Airborne Laser Mapping (NCALM) over the University of Houston [102]. The dataset covers the university campus and its neighborhood, containing 19 urban land cover/use classes. This dataset consists of MSL data, passive RGB imagery (5 cm GSD), passive hyperspectral data (48 bands at 380–1050 nm with 1m GSD), and rasterized ground truth for validation and is available upon request. Summary information about these benchmarks are reported in Table 6, whereas Figure 7 depicts these datasets.

## 4. Multispectral LiDAR Applications

**Figure 8:**
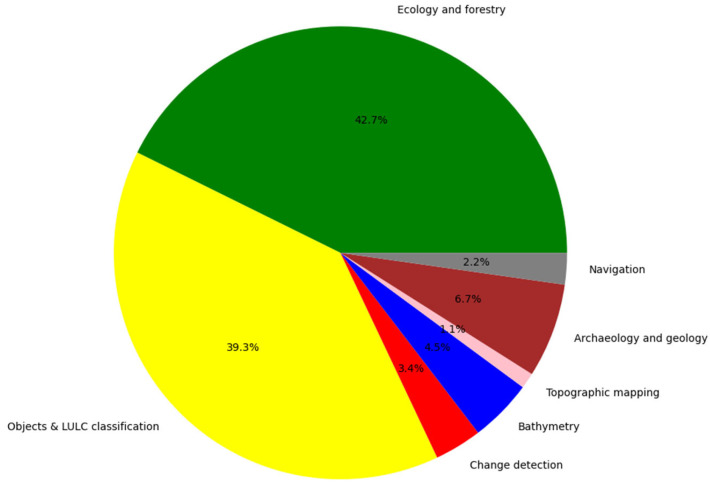
Subdivision of conducted research on MSL per application—based on Table A1.

As industrialization advances, the conventional methods of identifying and categorizing objects using optical images are no longer sufficient for achieving demanding precise outcomes [16,103]. With the ability to concurrently capture 3D point clouds in different wavelengths, MSL technology has attracted increasing attention for a variety of applications during the last decade. The applications of this revolutionary active remote sensing technology are comprised of the following: forest and urban trees/plants inventories, objects and LULC classification, change detection, bathymetry mapping and coastal zone management, topographic mapping, archaeology and geology, and last but not least, facilitating navigation systems. The following Section 4.1, Section 4.2, Section 4.3, Section 4.4, Section 4.5, Section 4.6, Section 4.7 provide detailed information on the applications mentioned above, which are based on reviewing 89 technical papers (see Table A1). Figure 8 shows the percentage frequency distribution of reviewed papers per application.

### 4.1. Ecology and Forestry

Most of the published research on MSL is in the domain of ecology and forestry (42.7%). Forest inventory plays a pivotal role in forest management. Traditionally, ecological studies have relied on laborious, time-consuming, and costly field visits to gather necessary information. However, remote sensing-based inventorying offers highly promising technology for these tasks without any destructive sampling and large-scale fieldwork. Thanks to the better canopy penetration capability of LiDAR sensors over optical ones, the use of laser scanners for accurate estimation of forest variables (such as tree height, basal area, stem volume, diameter at breast height, and above-ground biomass) has been an active research focus [104,105]. Nevertheless, traditional monochromatic laser scanners cannot capture enough information for tree and plant species classification, and a mix of tree species can even complicate that [28,80]. To date, passive multispectral optical sensors and their integration with airborne laser scanners have been widely used for forest tree species classification [106,107,108]. As different tree species reflect light at different wavelengths, modern MS laser scanners improve tree species identification accuracy compared to monochromatic LiDAR systems, particularly when tree species diversity is fairly high (about seven or more species) [13]. In MSL sensors, features describing the 3D structure of tree crowns as well as spectral information can be used for more detailed analysis of backscatters. Hence, the characterization of tree species, even identifying invasive ones, is one of the primary and most popular applications of multi-wavelength laser scanning [109]. Plant reflectance is high at NIR/SWIR wavelengths and low at the green wavelength due to their chlorophyll content, making a combination of the green laser channel and NIR/SWIR wavelength potentially useful for vegetation analysis. In a similar manner to the conventional Normalized Difference Vegetation Index (NDVI) derived from the visible red and NIR spectral bands, multiple wavelengths of MSL facilitate the calculation of NDVI/pseudo NDVI (pNDVI) or other vegetation indices. Tian et al. [110] employed an HSL with 64 wavelength channels (535 nm–850 nm with a 5 nm step), to classify six plant species using fusion of deep learning-based features and vegetation indices.

MSL technology also has the potential to enhance the accuracy of individual tree detection, especially in dense forests with clumped trees, which is quite often challenging using only geometric information [109]. This capability was first explored by Dai et al. [17], and they applied the mean shift segmentation method in a joint domain of spatial and spectral features. Also, the spectral information of MSL was utilized for refining under-segmented crown segments. Theirs results showed a noticeable improvement in dealing with clumped crowns compared to monochromatic wavelength laser scanning. In another study, according to the findings of Huo and Lindberg [111], incorporating intensity values in conjunction with a point density metric resulted in a noteworthy increase of up to 14% in F-scores.

Furthermore, MSL can also be helpful in the more accurate estimation of other parameters of trees. The research conducted by Gaulton et al. [70], utilizing SALCA dual-wavelength MSL, demonstrated improvement in the estimation of canopy cover, gap fraction, and leaf area index. Using three-wavelength Optech Titan LiDAR, Goodbody et al. [112] modeled three forest inventory attributes (i.e., Lorey’s height, gross volume, and basal area) as well as three overstorey species diversity characteristics, including Shannon index, Simpson index, and species richness. Their findings revealed that although the incorporation of intensity metrics yielded a modest enhancement in accuracy, the significance of these metrics becomes particularly pronounced when dealing with lower-resolution data in the context of 1 m and 2 m voxel models. The results of Maltamo et al. [113] substantiated the better efficiency of MSL in the prediction of forest canopy fuel parameters, including canopy fuel weight, canopy base height, biomass of living and dead trees, and height and biomass of the understory tree layer and site fertility. In 2023, Rana et al. [114] showed that MSL is superior to the combination of traditional monochromatic LiDAR and color–infrared image in monitoring seedling stands. In addition, the use of MSL makes the physiological and health condition analysis of vegetation possible [43,49,115] and furthermore enables a better understanding of periodic changes in carbon content [116]. Junttila [49] discovered that varying levels of leaf water content in Norway spruce seedlings exhibit distinct spectral responses while measured using terrestrial MSL. Their experiments demonstrated that the normalized ratio of two wavelengths, specifically at 905 nm and 1550 nm, holds significant utility in the estimation of leaf water content. Lately, Shao et al. [91] substantiated that HSL can also be helpful for more accurate wood–leaf separation, which mostly relies on monochromatic LiDAR.

### 4.2. Objects and LULC Classification

Accurate land use land cover classification plays an essential role in urban planning, monitoring climate changes, and ecosystem protection [117]. In the early studies of LULC classification, multispectral image data were used as the primary source of sensing Earth surface objects in order to facilitate more detailed object detection. Therefore, MSL is a new promising sensor for automated mapping of land cover [118]. The use of MSL technology allows for achieving 3D land cover classification at a finer scale using only MS point cloud data. MSL data have a comparable level of detail to aerial images, which are currently the primary data source in map updating. Several studies have confirmed that laser scanner intensity has merit in classifying urban land cover without the aid of passive multispectral images [119,120,121,122,123,124]. Chen et al. [116] observed that the spectral patterns of impervious surfaces (e.g., road, rooftops) and single-return vegetation (i.e., grass) have similar patterns in optical imagery. Using MSL, up to 70% overall accuracy can be achieved in land cover mapping solely based on intensity measurements [9]. On the other hand, incorporating both geometric and radiometric records, the accuracy could increase. Hence, MSL systems, integrating both spectral and geometric information, support the classification of point clouds and could have a vital role in nationwide mapping in the future [103]. Besides ecology, LULC mapping has attracted remarkable research attention based on MSL technology (39.3%).

### 4.3. Change Detection

With the rapid development of society, there are increasing demands for more precise monitoring of surface changes. Recently, the potential of automated change detection from multitemporal airborne MSL was explored for the first time by Matikainen et al. [125]. It was concluded that even small changes can be revealed by direct comparisons between height and intensity data from different dates. As a result of conducted research, MSL data could significantly contribute to increasing the level of automation in nationwide mapping, the frequency of its updates, and consequently improve the contents of topographic databases, which are currently mainly based on visual interpretation of the images [103,125,126].

### 4.4. Bathymetry

Generally, MSL instruments are not specifically tailored for hydrographic mapping, but as they encompass a green laser, they have exhibited bathymetric capabilities [127]. The first usage of MSL dates in this domain dates back to 2016 when Fernandez-Diaz et al. [128] mapped bathymetry by extracting DSM and intensity images of three channels as well as employing Mahalanobis distance and the maximum likelihood classifiers. Moreover, in the next year, using MSL point cloud data gathered by the Optech Titan sensor and also by extracting several geometric and radiometric features, Morsy et al. [129] classified water areas from land by employing a rule-based classification. In another study using a similar sensor, Yan et al. [130] mapped the water surface based on a 3D maximum likelihood classifier. These studies demonstrated that for mapping the water bodies’ areas, MSL is especially more beneficial than conventional monochromatic LiDAR systems. Furthermore, MSL could facilitate the monitoring of hydromorphological status by estimating some critical indicators such as water depth, leaf area index, and chlorophyll content [131].

### 4.5. Topographic Mapping

Recently, Ali et al. [132] proposed the idea of generating DTM from MSL data. They extracted DTM from each channel of the Optech Titan MSL sensor separately and made a comparison between them. They also examined the potential of four different ground-filtering algorithms, including Adaptive TIN (ATIN), Elevation Threshold with Expansion Window (ETEW), progressive morphological algorithms, and maximum local slope using LiDAR open-source ALDPAT v.1.0 software. Their results showed that in the water area, the slope-based and ETEW methods performed well for the third channel. However, for the other two channels, the morphology-based method yielded better results.

### 4.6. Archaeology and Geology

One of the interesting and firstly introduced application areas of MSL is archaeological prospection [73]. In 2006, Wehr et al. [69] detected the damaged areas of building surfaces caused by enhanced moisture content and/or vegetation using the designed four-wavelength MSL. Shao et al., 2019 [94] employed a designed AOTF-HSL to preserve historical timber buildings. They classified building ages and wood species using the spectral information of an eye-safe 81-channel HSL. Additionally, MSL data can assist geological studies and mining operations. Hartzell et al. [133] utilized intensity images acquired from an integrated system that included the RIEGL VZ-400 TLS (NIR) and Nikon D700 camera (RGB) to distinguish between four different types of rock. Using AOTF-HSL, Shao et al., 2019 [87], managed to identify four-type coal/rock specimens. Newly, Sun et al. [44] showed that spectral profiles collected by hyperspectral LiDAR can effectively reveal the ore species, especially those in the SWIR range. According to their experiments, HSL has demonstrated encouraging capability for geological material detection and classification and furthermore for tunnel modeling and also mineral disaster prevention applications.

### 4.7. Navigation

The feasibility of HSL systems for autonomous vehicle perception was recently explored by Taher et al. [134]. A frame-based single photon-sensitive HSL with 30 spectral channels ranging from 1200 to 1570 nm was developed for this purpose. Their results demonstrated that spectral information from an HSL can accelerate scene recognition accuracy in a complicated road environment from 50% to 94% with two channels and 30 channels, respectively. Furthermore, Jiang et al. [93] developed an intensity calibration-free method to aid point cloud matching in SLAM. Their method is based on designing an HSL that collects intensity data in eight wavelengths at the same incident angle and range, and subsequently, computing spectral ratio value vectors between consecutive laser scans, and finally applying them in point cloud matching. So, their method improved the accuracy of LiDAR SLAM positioning by combining LiDAR’s intensity information with its range measurements.

## 5. Discussion

According to Section 4.1, Section 4.2, Section 4.3, Section 4.4, Section 4.5, Section 4.6, Section 4.7, MSL technology exhibits a diverse range of applications, spanning from ecology and forestry to navigation. The incorporation of additional spectral information alongside geometric data presents invaluable opportunities for deriving new spectral features, particularly vegetation, water, and built-up indices. These capabilities open avenues for new opportunities in various applications, including but not limited to enhancing plant/tree species classification, enabling more precise forest inventory assessments, conducting physiological and health condition analyses, generating fine-grained 3D urban maps, automating change detection processes, improving the accuracy of water surface mapping, achieving detailed DTM and DSM separation, preserving historical buildings, detecting and classifying geological materials, supporting autonomous driving, and facilitating point cloud matching in SLAM. Nonetheless, challenges persist in the widespread implementation of MSL technology. The foremost hurdle across numerous applications involves the judicious selection of suitable spectral bands tailored to specific applications and existing objects. Additionally, a prevalent challenge encountered in MSL applications is the mitigation of systematic radiometric strip differences, necessitating meticulous attention to proper radiometric calibration processes [73]. Furthermore, the requirement for detecting several hundred photons per wavelength channel is imperative to attain a high level of accuracy in navigation applications [134]. Comprehensive insights into the potentials, opportunities, and challenges of MSL technology for each application are meticulously outlined in Table 7.

## 6. Conclusions

In the last two decades, geometrical information from LiDAR has been actively combined with passive multispectral information from optical images to achieve more accurate results. This paper has demonstrated how multispectral laser scanning can unlock more precise mapping with respect to the use of a single-data source. By meticulously analyzing existing research, it is revealed that MSL technology opens new doors across various application domains in the field of remote sensing and photogrammetry. The paper delves into seven key applications of MSL systems, encompassing “ecology and forestry”, “objects and LULC classification”, “change detection”, “bathymetry”, “topographic mapping”, “archaeology and geology”, and “navigation”. Each application is comprehensively examined, providing insights into their potentials, opportunities, and challenges. Due to wider application possibilities, active MSL/HSL systems provide new opportunities for fine-grained 2D and nD mapping. Therefore, MSL/HSL technology, by reducing the discussed challenges associated with common data fusion approaches, is a compelling alternative to existing multi-data source approaches for fine mapping. Thus, MSL is expected to quickly adapt to academic and industrial societies as a single-data source solution. The major limitation to this adaptation is the cost of the systems, since they might be multiple times that of monochromatic LiDAR systems. In spite of this, the commercial development of multi-wavelength laser scanners can facilitate the use of this technology. Even so, HSL and MSL technologies have paved the way for nD geometric–radiometric data acquisition and consequently more reliable 2D/nD mapping. Therefore, MSL and HSL data processing are expected to remain active areas of research in the coming years. Upon comprehensively reviewing past research on contemporary MSL/HSL technology (see Table A1), we found that still there are a lot of opportunities for further investigations, as follows:

- The majority of MSL/HSL systems are designed for experimental purposes. Notably, there is currently no commercially available HSL system at the moments. Consequently, there is a need to introduce new MSL/HSL systems to the market.
- Due to the promising capabilities of NASA’s GEDI spaceborne LiDAR (launched in late 2018) in canopy height and aboveground biomass estimation, satellite-based MSL can be also anticipated in the near future [135].
- Given that spectral information constitutes the primary advantage of MSL technology over monochromatic LiDAR, there is an increased demand for precise radiometric calibration [136]. Therefore, it is worthwhile to consider the incorporation of a radiometric calibration component in the design of the new generation of MSL/HSL systems.
- A notable limitation of Titan data is the presence of inhomogeneity within the point clouds, as significant discrepancies in the data between the across-track and along-track directions are visible [137]. Given that the 3-wavelength Optech Titan data are currently the most commonly utilized data in various studies, it becomes evident that there is a demand for the development and exploration of new commercial MSL/HSL systems with enhanced specifications in the near future. To address the mentioned issue and achieve a more uniform point spacing, upcoming MSL systems can consider either reducing the aircraft speed or increasing the scan frequency [137].
- Multispectral LiDAR instruments ought to be both cost-effective and compact in size, thereby facilitating their adoption into academic and industrial domains.
- The majority of SC-based HSL systems currently feature fewer than 10 spectral channels. Therefore, there is a need for the introduction of new HSL systems that offer a broader range of spectral information. Overcoming eye-safety issues is a primary consideration in this context.
- More attempts should be made for directly processing 3D MSL/HSL point clouds instead of considering rasterized data form.
- Benchmark datasets in MSL/HSL for scientific purposes, especially those with ground truth data, are still lacking.
- During recent years, forestry and LULC mapping have received by far the most attention from scholars. More studies are needed to be dedicated to the other mentioned applications of MSL, especially archaeology, navigation, and change detection.
- Multispectral laser scanning is expected to yield a broader spectrum of applications, such as extending to precision agriculture, disaster risk management, distinguishing pollution in environment, and detecting obscured targets [136].
- Increased attention should be directed towards thorough exploration of the potential opportunities of HSL systems.

## Figures and Tables

**Table 1:** The search strategy for screening relevant papers in the domain of MSL.

Platform	Search Query	Number of Found Papers
Scopus	“multispectral LiDAR” OR “multi-wavelength LiDAR” OR “bispectral LiDAR” OR “dual-wavelength LiDAR” OR “hyperspectral LiDAR” OR “multispectral laser” OR “multi-wavelength laser” OR “bispectral laser” OR “dual-wavelength laser” OR “hyperspectral laser” OR “multispectral light detection and ranging” OR “multi-wavelength light detection and ranging” OR “bispectral light detection and ranging” OR “dual-wavelength light detection and ranging” OR “hyperspectral light detection and ranging”	401
Web of Science		278

**Table 2:** Multispectral passive vs. active sensors.

	Multispectral Passive Sensors (Cameras)	Multispectral Active Sensors (LiDAR)
Advantages	✓ Less computational burden, especially for large-scale mapping ✓ Easier interpretability ✓ Benefiting from numerous automated processing methods (esp. deep learning) ✓ Greater data availability ✓ Mostly having higher spectral resolution ✓ Broader coverage ✓ Some free data (e.g., Sentinel or Landsat series)	✓ Incorporating both spectral and precise 3D geometrical information ✓ Shadowless intensity images (active sensor) ✓ Without relief displacement error ✓ Independent data acquisition of weather conditions ✓ Feasibility of 3D point cloud classification and object recognition ✓ More accurate results (3D view) ✓ Having higher spatial resolution in general
Disadvantages	🗴 Lack of geometrical information 🗴 Higher misinterpretation and misclassification error (2D view) 🗴 Shadow probability (passive sensor) 🗴 Relief displacement problem 🗴 Measuring the spectral response as a function of distance (e.g., depth into a forest canopy) is impossible	🗴 More computationally expensive 🗴 More sophisticated data interpretation (irregular distribution) 🗴 Attracted much less attention in developing automated processing techniques 🗴 Lower coverage rate

**Table 3:** Currently available passive multispectral sensors in remote sensing. Abbreviations: GSD: Ground Sample Distance; MSI: Multispectral Instrument; OLI: Operational Land Imager; WV110: WorldView-110 camera.

Passive Multispectral Sensor	Platform	Operator	Spectral Bands	GSD (m)	Altitude (km)	Stereo	Revisit (Day)
DJI P4 MS [29]	Drone	DJI	5	0.095	Flexible	Yes	Flexible
Parrot Sequoia [30]	Drone	Parrot	4	0.05	Flexible	Yes	Flexible
Sentera 6X MS [31]	Aerial	Sentera	5	0.026	Flexible	Yes	Flexible
S2A MSI [32]	Sentinel-2A	ESA	13	10 (B2–B4 & B8) 20 (B5–B7 & B12–B13) 60 (other bands)	790	No	10
S2B MSI [32]	Sentinel-2B	ESA	13	10 (B2–B4 & B8) 20 (B5–B7 & B12–B13) 60 (other bands)	790	No	10
OLI-1 [33]	Landsat 8	NASA	9	30	705	No	16
OLI-2 [34]	Landsat 9	NASA	9	30	705	No	16
ASTER [35]	Terra	NASA/METI	14	15	705	Yes	16
MSI [36]	Pleiades-1	Astrium	4	2.8	695	Yes	1
WV110 [37]	WorldView-2	MAXAR	8	1.84	773	Yes	1.1
WV110 [38]	WorldView-3	MAXAR	8	1.25	617	Yes	1

**Table 4:** List of some single-wavelength LiDAR sensors that can be used in multi-sensor MSL systems. PRF = pulse repetition frequency; A = airborne; T = terrestrial.

Wavelength	LiDAR Sensor	Producer	Platform	Beam Divergence [mrad]	Looking Angle [°]	PRF [kHz]
Green	VQ-840-G [56]	RIEGL	A & T	1.0–6.0	40	≤200
	VQ-820-G	RIEGL	A	1	1–60	≤520
	Aquarius [57]	Optech	A	1	0–±25	33, 50, 70
Red	HDS6100	Leica	T	0.22	360 × 310	NA
NIR	VQ-580	RIEGL	A	0.2	60	≤380
	VUX-1HA [58]	RIEGL	A & T	0.5	360	≤1000
	MiniVUX-3 UAV [59]	RIEGL	A	0.8	360	≤300
	Gemini [60]	Optech	A	0.25 & 0.8	0–50	33–167
	ALTM Galaxy T1000 [61]	Optech	A	0.25	10–60	50–1000
	Pegasus [62]	Optech	A	0.25	±37	100–500
	TerrainMapper [63]	Leica	A	0.25	20–40	≤2000
	CityMapper [64]	Leica	A	0.25	40	≤700
	FARO S120	FARO	T	0.19	360 × 305	97
	Trimble TX5 [65]	Trimble	T	0.19	360 × 300	97
SWIR	VQ-480i [66]	RIEGL	A	0.3	60	≤550
	LMS-Q680i	RIEGL	A	≤0.5	60	≤400
	FARO X330	FARO	T	0.19	360 × 300	97
	Orion [67]	Optech	A	0.25	10–50	0.05/0.06

**Table 6:** Summary of released MSL/HSL benchmark datasets.

Dataset, Year	Producer	Data Type	LiDAR System	Wavelength	Area Type	Area of Coverage	Auxiliary Data
ISPRS WG III/5, 2015	ISPRS WG III/5 and Teledyne Optech	3D	Optech Titan	SWIR, NIR, and green	Natural coastal	Tobermory (ON, Canada)	NA
IEEE GRSS, 2018	NCALM	2D	Optech Titan	SWIR, NIR, and green	Urban	University of Houston campus and its neighborhood	RGB and hyperspectral image

**Table 7:** Summary of the challenges, potentials, opportunities, and challenges of MSL technology.

Application	Potentials	Opportunities	Challenges
Ecology and forestry	Gathering spatial-spectral information from canopies and under canopies Vegetation indices	Aiding plant/tree species classification Increasing accuracy of individual tree segmentation and wood–leaf separation Physiological and health condition analyses More accurate estimation of other tree parameters	Better exploit plant reflectance in the different wavelengths Improve characterization of single species
Objects and LULC classification	Incorporating spectral features Built-up indices	Fine-grained 3D uraban mapping Facilitating detecting ground-level objects Proposing single-data source solution	Understand relationships between wavelengths and needed classes A proper radiometric calibration is necessary to reduce systematic differences between radiometric strips
Change detection	More precise automated monitoring of surface changes	Replace visual interpretation of multi-temporal images	Upscaling, costs, appropriate radiometric calibration
Bathymetry	Richer spectral information Water indices	More accurate water surface mapping Monitoring of hydromorphological status	Dealing with other challenging shore areas (e.g., delta wetland, rocky shore, and shore with land depression)
Topographic mapping	Improved DTM generation by using spectral information	Detailed DTM/DSM separation	Filtering areas with water
Archaeology and geology	Different reflectance behavior of object at different wavelengths	Preserve historical buildings Detecting the damaged areas of building Geological material detection and classification Supporting mining operations Tunnel modeling Mineral disaster prevention	Appropriate wavelength selection with respect to the actual surface status Systematic radiometric strip differences should be reduced by a proper radiometric calibration process
Navigation	Requiring less illumination power Less prone to motion blur Providing useful information of material-specific spectral signatures	Autonomous driving Assisting point cloud matching in SLAM Higher scene recognition accuracy in a complicated road environment	Detection of multiple hundreds of photons per wavelength channel is required for achieving high accuracy Optimal channel selection should be carried out

## Data Availability

Data is contained within the article.

## Appendix A

**Table A1:** Exhaustive review of technical papers on MSL data processing, organized by publication date. Abbreviations: ^P^ = Point cloud; ^I^ = Image; DBM = Deep Boltzmann Machine; NDFI = Normalized Difference Feature Index; PRI = Photochemical Reflectance Index; MNDWI = Modified Normalized Difference Water Index; CHM = Canopy Height Model; LDA = Linear Discriminant Analysis; SVM = Support Vector Machine; RF = Random Forest.

Publications	Data Sources	Application	Feature(s) Type	Method(s)
Hakula et al., 2023 [68]	HeliALS-TW	Tree species classification	Several geometric, radiometric and return type features	^P^ Object-based RF
Rana et al., 2023 [114]	Optech Titan	Monitoring seedling stands	Geometric, intensities	^P^ Linear regression
Axelsson et al., 2023 [138]	VQ-1560i-DW	Tree species classification and stem volumes estimation	Several geometric, radiometric and return type features	^P^ LDA and k-nearest neighbors models
Shao al., 2023 [90]	AOTF-HSL	Persimmon tree components classification	Intensities, average reflectance, CI red edge, NDVI, NDRE	^P^ RF, SVM, and backpropagation neural network methods
Xiao et al., 2022 [119]	Optech Titan	LULC classification	Z, intensity, and SVD-based and deep features	^P^ Improved RandLA-Net
Tian et al., 2022 [110]	Designed HSL	Plant species classification	Deep features and vegetation indices	^I^ VI-CNN
Zhang et al., 2022 [121]	Optech Titan	LULC classification	Deep	^P^ MPT + network
Taher et al., 2022 [134]	Designed HSL	Autonomous vehicle perception	Intensities and geometric	^I^ RF
Li et al., 2022 [139]	Optech Titan	LULC classification	Deep	^P^ AGFP-Net
Mielczarek et al., 2022 [109]	VQ-1560i-DW	Invasive tree species identification	Statistical features of intensities, GNDVI	^P^ Object-based RF, gradient boosting, Xgboost, and SAMME.R
Luo et al., 2022 [10]	Optech Titan	LULC classification	DSM, intensities, pNDVIs	^P^ Decision tree
Sun et al., 2022 [44]	Designed HSL	Objects (manufacturing materials, plants and ore species) classification	Intensities	^P^ Rule-based
Morsy et al., 2022 [8]	Optech Titan	LULC classification	Z, pNDVIs	^P^ Rule-based
Lindberg et al., 2021 [99]	Optech Titan	Tree species classification	Mean and std of intensities	^I^ LDA
Shao et al., 2021 [91]	AOTF-HSL	Wood-leaf separation	Intensity ratio, first derivative of spectral reflectance	^P^ Rule-based
O. Ali et al., 2021 [132]	Optech Titan	DTM generation	Z and intensities	^P^ Adaptive TIN, elevation threshold, progressive morphological algorithms, and maximum local slope
Shi et al., 2021 [122]	Optech Titan	LULC classification	Multi-scale statistical features and NDFIs	^P^ SVM
Ghaseminik et al., 2021 [123]	Optech Titan	LULC classification	Intensity images, DTM, DSM, n DSM, slope, aspect, eigen value-based features,	^I^ Object-based RF
Zhao et al., 2021 [140]	Optech Titan	LULC classification	Deep	^P^ FR-GCNet
Jing et al., 2021 [141]	Optech Titan	LULC classification	Deep	^P^ Squeeze-and-Excitation (SE) PointNet++
Pan et al., 2020 [142]	Optech Titan	LULC classification	Deep	^I^ CNN
Imangholiloo et al., 2020 [143]	Optech Titan	Forest inventory	CHM, pNDVI, intensities and its ratio and statistical features	^I^ Object-based RF
Jiang et al., 2020 [92]	AOTF-HSL	Points cloud classification	Intensity ratios	^P^ Rule-based classification
Huo & Lindberg, 2020 [111]	Optech Titan	Individual tree detection	Maximum height, point density, vegetation ratio, and average intensities	^P^ Local height maximum filter and similarity maps
Matikainen et al., 2020 [55]	Combination of SPL100 and Optech Titan	LULC classification	Geometric-spectral statistical features, Geometric-spectral GLCM-based and GLDV-based textural features, pNDVI, brightness, pNDBI, intensity ratios	^I^ Object-based RF
Maltamo et al., 2020 [113]	Optech Titan	Prediction of forest canopy fuel parameters	Geometric-spectral statistical features, echo class proportion, sum of intensities, ratio of two channels	^I^ LDA and linear regression
Goodbody et al., 2020 [112]	Optech Titan	Forest inventory and diversity attribute modelling	Geometric-spectral statistical features, NDFI, sum of all channels, ratios of two channels and CVI	^P^ RF
Li et al., 2020 [19]	Optech Titan	Buildings extraction	Deep	^P^ Graph Geometric Moments (GGM) convolution
Jiang et al., 2020 [93]	AOTF-HSL	SLAM points cloud matching	Spectral ratio	^P^ Iterative Closest Point (ICP)
Yan et al., 2020 [144]	Optech Titan	Predicting forest attributes	Geometric-spectral statistical features and pNDVIs	^P^ RF
Junttila et al., 2019 [145]	Integration of FARO X330 and Trimble TX5	Detecting tree infestation	Spectral statistical features and density bandwidth	^P^ Regressions and linear discriminant analysis
Kukkonen al., 2019 [28]	Optech Titan	Tree species classification	Geometric statistical features, channel intensities, sum of two channels, sum of all channels, ratios of two channels	^I^ k-nearest neighbors
Jiang al., 2019 [22]	AOTF-HSL	Vegetation red edge parameters extraction	Intensities	^P^ First-order differential of the spectral reflectance
Shao et al., 2019 [87]	AOTF-HSL	Coal/rock classification	Intensities	^P^ Naive Bayes, logistic regression, and SVM
Pan et al., 2019 [124]	Optech Titan	LULC classification	Deep, intensities, GDVI, GRVI, GNDVI, MNDWI, Geometric-spectral GLCM	^I^ DBM-based deep feature extraction, PCA-based and RF-based low level feature selection, SVM
Wang and Gu, 2019 [146]	Optech Titan	LULC classification	Geometric-spectral eigen values	^P^ Tensor manifold discriminant embedding and SVM
Pilarska et al., 2019 [147]	VQ-1560i-DW	Urban tree classification	Spectral statistical features and pNDVI	^P^ SVM
Kukkonen al., 2019 [12]	Optech Titan	Tree species classification and predicting species’ volume proportions	Intensity, geometric-spectral statistical features, channels ratios, binary sum of two channels, and density	^I^ LDA model
Matikainen et al., 2019 [103]	Optech Titan	Change detection	DSM, intensities, pNDVI, and NDBI	^I^ Object-based RF and rule-based classification
Shao et al., 2019 [94]	AOTF-HSL	Architectural heritage preservation	Intensities	^P^ Naive Bayes and SVM
Junttila et al., 2018 [49]	Integration of Leica HDS6100, FARO S120 and FARO X330	Estimating leaf water content	Spectral statistical features, normalized difference indices and spectral ratios	^P^ Simple linear regression
Karila et al., 2018 [118]	Optech Titan	LULC classification and road detection	Spectral statistical features, intensity ratios, GLCM homogeneity, ratios of two channels, PNDVI, and NDBI) and DSM features (std, GLCM homogeneity, and quartiles difference)	^I^ Object-based RF
Ekhtari et al., 2018 [7]	Optech Titan	LULC classification	nDSM and intensities	^P^ SVM and rule-based classification
Dai et al., 2018 [17]	Optech Titan	Individual tree detection	Geometric features and intensities	^P^ Mean shift segmentation and SVM
Pilarska, 2018 [148]	VQ-1560i-DW	LULC classification and road detection	nDSM, intensities, and GNDVI	^I^ Rule-based classification
Chen et al., 2018 [116]	Optech Titan	Quantifying the carbon storage in urban trees	nDSM, intensity images, pNDVI, and pNDWI	^I^ SVM, watershed segmentation, and allometry-based linear regression
Huo et al., 2018 [149]	Optech Titan	LULC classification	Intensities, nDSM, pNDVI, morphological profiles, and a novel hierarchical morphological profiles	^I^ SVM
Axelsson et al., 2018 [150]	Optech Titan	Tree species classification	Statistical features of heights and intensities	^P^ LDA model
Dalponte et al., 2018 [11]	Optech Titan	Predicting forest stand characteristics	Statistical features of heights and intensities	^P^ Ordinary least squares regression
Yan et al., 2018 [130]	Optech Titan	Water surface mapping	Elevation, elevation variation, intensity, intensity variation, number of returns and NDFIs	^P^ maximum likelihood
Kaszczuk et al., 2018 [151]	MSL	Plants condition analysis	NA	NA
Goraj et al., 2018 [131]	VQ-1560i-DW	Identifying hydromorphological indicators	Statistical features of intensities, and NDVI	^I^ Regression
Chen, 2018 [152]	Optech Titan	LULC classification	Deep	^I^ 3D CNN
Matikainen et al., 2017 [16]	Optech Titan	LULC classification	41 features based on DSM, DTM, and intensityimages of three channels	^I^ Object-based RF
Yu et al., 2017 [153]	Optech Titan	Tree species classification	145 features based on Z, density, number of returns, 2D and 3D convex hull, spatial statistical features	^P^ Object-based RF
Morsy et al., 2017 [154]	Optech Titan	LULC classification	Z, and pNDVIs	^P^ Gaussian decomposition-based clustering
Karila et al., 2017 [18]	Optech Titan	Road mapping	Mean, std, quantiles and ratios of channels, pNDVI, DSM differences	^I^ Object-based RF
Morsy et al., 2017 [129]	Optech Titan	Land/water classification	Elevation difference and roughness, intensity coefficient of variation (ICOV) and intensity density (ID), point density (PD) and multiple returns (MR)	^P^ Rule-based classification
Matikainen et al., 2017 [126]	Optech Titan	LULC classification, road mapping, and change detection	DSM, DTM, intensity images	^I^ Object-based RF
Morsy et al., 2017 [97]	Optech Titan	LULC classification	Z/DSM and three pNDVIs	^P^ Maximum likelihood and rule-based classification
Budei et al., 2017 [13]	Optech Titan	Tree species classification	pNDVIs, intensities, CHM	^I^ RF
Teo and Wu, 2017 [14]	Optech Titan	LULC classification	nDSM, intensities, NDFIs, curvatures	^I^ Object-based SVM
Morsy et al., 2016 [155]	Optech Titan	LULC classification	NDWI, pNDWI, MNDWI	^P^ Rule-based classification
Ahokas et al., 2016 [137]	Optech Titan	Tree species and LULC classification	DSM and several statistical features of intensity images	^I^ Object-based RF
Hopkinson et al., 2016 [77]	Integration of Aquarius and Orion, Gemini, and Optech Titan	Forest land surface classification and vertical foliage partitioning	Intensities	^I^ Minimum distance, maximum likelihood and parallelepiped classification routines
Nabucet et al., 2016 [156]	Optech Titan	Urban vegetation mapping	CHM and NDFI	^I^ Object-based rule-based classification
Bakuła et al., 2016 [157]	Optech Titan	LULC classification	nDSM, intensities, morphological granulometric features	^I^ Integration of maximum likelihood and rule-based classification
Fernandez-Diaz et al., 2016 [128]	Optech Titan	LULC classification, bathymetry mapping, thick vegetation canopies mapping	DSM and intensities	^I^ Mahalanobis distance and the maximum likelihood
ZOU et al., 2016 [158]	Optech Titan	LULC classification	pNDVI, elevation difference ratio_green, ratio_count	^I^ Object-based rule-based classification
Junttila et al., 2016 [115]	Integration of FARO X330 and Leica HDS6100	Measuring leaf water content	Spectral statistical features, ratios of intensities, and NDFI	^P^ Simple linear regression
Morsy et al., 2016 [9]	Optech Titan	LULC classification	DSM and intensities	^I^ Maximum Likelihood
Matikainen et al., 2016 [125]	Optech Titan	Change detection	Spectral statistical features, intensity ratios, NDVI, DSM and its statistical features, homogeneity of DSM	^I^ Object-based RF
Miller et al., 2016 [159]	Optech Titan	LULC classification	Height, return number, intensities, pNDVI	^I^ Maximum likelihood
Hakala et al., 2015 [160]	Designed HSL	Monitoring pine chlorophyll content	MCARI750, MSR2, SR6 and NDVI	^P^ Regression
Bakuła, 2015 [161]	Optech Titan	LULC classification	nDSM, intensities	^P^ Terrasolid software
Shi et al., 2015 [162]	MWCL	LULC classification	Vegetation indices (NDVI, GNDVI, and SRPI)	^P^ SVM
Junttila et al., 2015 [163]	Full waveform HSL	Trees drought detection	Spectral statistical features, NDVI, and a modified water index	^P^ Rule-based classification
Lindberg et al., 2015 [74]	Integration of VQ-820-G and VQ-580, VQ-820-G and VQ-480i	Tree species classification	nDSM and maximum reflectance of first return for each wavelength	^I^ Rule-based classification
Wichmann et al., 2015 [120]	Optech Titan	LULC classification	Intensities and pNDVI	^P^ Mahalanobis distance
Gong et al., 2015 [78]	Wuhan MSL	LULC classification	Intensities	^I^ SVM
Hartzell et al., 2014 [133]	Integration of RIEGL VZ-400 TLS and Nikon D700 camera	Rock type identification	Intensities	^I^ Minimum distance
Wallace et al., 2014 [43]	TCSPC	Recovery of arboreal parameters	Intensities, NDVI, and PRI	^I^ Reversible jump Markov chain Monte Carlo
Gaulton et al., 2013 [164]	SALCA	Estimating vegetation moisture content	NDFIs, ratios of intensities, NDWI and moisture stress index	^P^ Major axis regression
Briese et al., 2013 [73]	Integration of VQ-820-G and VQ-580, VQ-820-G and VQ-480i	Archaeological prospection	Intensities	^I^ Visual interpretation
Wang et al., 2013 [54]	Pegasus and Q680i	LULC classification	Z, echo width, and intensities	^I^ SVM
Wallace et al., 2012 [80]	SELEX GALILEO	Forest canopy parameter estimation	NDVI and PRI	^I^ MCMC and RJMCMC simulation
Woodhouse et al., 2011 [79]	Designed MSL	Measuring plant physiology	Z, PRI and NDVI	^P^ TREEGROW model
Gaulton et al., 2010 [70]	SALCA	Measurement of canopy parameters	Intensities	^P^ Rule-based
Wehr et al., 2006 [69]	BAM	Inspection of building surfaces	Intensities, NDVI, NDMI	^I^ Object-based classification with commercial software
